# Lower need for allogeneic blood transfusion after robotic low anterior resection compared with open low anterior resection: a propensity score-matched analysis

**DOI:** 10.1007/s11701-023-01571-5

**Published:** 2023-03-28

**Authors:** Erik Wiklund, Johan Carlander, Philippe Wagner, Malin Engdahl, Abbas Chabok, Maziar Nikberg

**Affiliations:** 1 Colorectal Unit, Department of Surgery, Västmanland Hospital Västerås, 72189 Västerås, Sweden; 2 Colorectal Unit, Department of Surgery, Centre for Clinical Research of Uppsala University, Västmanland Hospital Västerås, Västerås, Sweden

**Keywords:** Rectal cancer, Robotic surgery, Blood transfusion, Anterior resection

## Abstract

Robotic low anterior resection (R-LAR) for rectal cancer may decrease estimated blood loss compared with open low anterior resection (O-LAR). The aim of this study was to compare estimated blood loss and blood transfusion within 30 days after O-LAR and R-LAR. This was a retrospective matched cohort study based on prospectively registered data from Västmanland Hospital, Sweden. The first 52 patients operated on using R-LAR for rectal cancer at Västmanland Hospital were propensity score-matched 1:2 with patients who underwent O-LAR for age, sex, ASA (American Society of Anesthesiology physical classification system), and tumor distance from the anal verge. In total, 52 patients in the R-LAR group and 104 patients in the O-LAR group were included. Estimated blood loss was significantly higher in the O-LAR group compared with R-LAR: 582.7 ml (SD ± 489.2) vs. 86.1 ml (SD ± 67.7); *p* < 0.001. Within 30 days after surgery, 43.3% of patients who received O-LAR and 11.5% who received R-LAR were treated with blood transfusion (*p* < 0.001). As a secondary post hoc finding, multivariable analysis identified O-LAR and lower pre-operative hemoglobin level as risk factors for the need of blood transfusion within 30 days after surgery. Patients who underwent R-LAR had significantly lower estimated blood loss and a need for peri- and post-operative blood transfusion compared with O-LAR. Open surgery was shown to be associated with an increased need for blood transfusion within 30 days after low anterior resection for rectal cancer.

## Introduction

Rectal cancer accounts for about 700,000 new cases each year throughout the world [1]. The treatment of rectal cancer includes combinations of radiotherapy, chemotherapy, and surgery depending on the tumor characteristics and individual patient’s needs. Recent advances in minimally invasive procedures include laparoscopic and, more recently, robot-assisted surgery. Robot-assisted low anterior resection (R-LAR) has been reported as feasible and to have non-inferior oncological outcomes compared with laparoscopic and open surgery for the treatment of rectal cancer [2–6]. Some previous studies have shown lower estimated blood loss after laparoscopic surgery compared with open surgery [7, 8], whereas another study reported similar blood loss [9]. Only one study has reported on estimated blood loss and the need for peri-operative blood transfusion when comparing open low anterior resection (O-LAR) and R-LAR [10]. This study showed a significantly greater decrease in hemoglobin level and higher estimated blood loss after O-LAR than after R-LAR.

The prevalence of pre-operative anemia is high in patients with colorectal cancer [11–14]. A meta-analysis by Wilson et al. showed that pre-operative anemia is associated with inferior long-term overall survival and disease-free survival in patients with rectal cancer [15]. Anemia in patients with colorectal cancer is often accompanied by iron deficiency which, if treated, leads to improved hemoglobin level and reduced need for peri-operative blood transfusion [16]. Patients with and without anemia sometimes need blood transfusion in the peri-operative period, but blood transfusions can cause both infectious and non-infectious adverse events. Infectious complications are rare, but non-infectious reactions occur in 1:373 transfusions [17]. The long-term effects of blood transfusions on disease-free survival remain unclear; some studies have reported worse outcomes [14, 18–21], while other studies have reported equivalent long-term outcomes [22–25].

Regardless, there is broad consensus that the use of blood products should be kept at the lowest level possible. The aim of this study was to compare the estimated blood loss and frequency of blood transfusion within 30 days after O-LAR and R-LAR.

## Methods

Robot-assisted colorectal surgery was introduced at Västmanland Hospital in 2016. All of the first 52 consecutive patients who underwent R-LAR were identified and included as index patients. The referents were identified from the same regional population-based rectal cancer registry in the county of Västmanland. This registry holds prospectively collected detailed data from the pre-, peri-, and post-operative periods. To facilitate data collection and to reduce confounding bias, index patients were matched 1:2 with patients who underwent O-LAR using a propensity score (PS) based on the variables age, sex, American Society of Anesthesiology (ASA) grade, and tumor level (distance from the anal verge). The included patients underwent open surgery between June 2000 and March 2020, and robot-assisted surgery between March 2016 and March 2020.

Data for hemoglobin level on post-operative days 1–5, tumor distance from the anal verge, tumor-node-metastasis (TNM) stage, pre-operative radiotherapy, pre-operative chemotherapy, comorbidities, post-operative C-reactive protein (CRP) level, duration of surgery, complications within 30 days, reoperations, estimated blood loss, and mortality were gathered from the local registry. The number of transfused units and transfusion volume were determined from the local registry and crosschecked with the hospital’s electronic transfusion records. Estimated blood loss is routinely recorded at the end of surgery and is determined by the surgeon in charge and the anesthesiologist, but no precise measurements of blood loss were made. Data for pre-operative hemoglobin level and use of iron supplement were gathered retrospectively from health records. All complications were categorized as infectious, surgical or medical. No patients were lost to follow-up.

### Surgery

Low anterior resection was performed using either partial or total mesorectal excision by a few experienced colorectal surgeons. For both R-LAR and O-LAR, coloanal or colorectal side-to-end anastomoses were constructed using the sigmoid or descending colon. Central ligation of the inferior mesenteric artery 2 cm from the aorta or ligation of the superior rectal artery close to the origin of the left colic artery was routinely performed.

During open surgery, sharp dissection following the embryological planes was performed using monopolar diathermy. In most cases, the splenic flexure was mobilized. Robotic surgery was performed using the da Vinci Xi system. The dissection was performed using diathermy scissors in the embryological planes and following the same oncosurgical principles as in open surgery. Two pelvic drains were placed routinely during open surgery and one drain during robotic surgery. All patient data including surgical technique, complications, blood loss, and transfusion during surgery were registered by the surgeon at the time of the surgery.

### Statistical analysis

PS was generated using logistic regression and evaluated by examining the balance using standardized differences. Matching was then performed by stratifying PS into quintiles and randomly selecting two patients treated with O-LAR for each patient treated with R-LAR within the same PS stratum. Summary statistics for patient characteristics, clinically relevant outcomes, estimated blood loss, and number of blood transfusions were arranged according to the surgery type [and expressed as counts, percentages, mean, median, standard deviation (SD), minimum and maximum].

Continuous data are presented as the mean with SD or median with range. Categorical data were compared using Chi-square or Fisher’s exact test. Conditional logistic regression stratified for PS-quintile groups (CLR) was used to quantify differences in the primary outcome; need for transfusion within 30 days, between O-LAR and R-LAR and to adjust for additional confounders not accounted for by the PS matching. Adjustment variables were identified according to the disjunctive cause criterium, in short, that means mainly selecting variables that are a cause of treatment, outcome or both [26]. However, to avoid overfitting bias by adhering to the “one in ten rule” [27], stating that there should be a minimum of ten events for each variable included in the model, adjustment variables were limited to those assumed to induce the greatest confounding effect. Secondary sensitivity analyses including only one potential risk factor at the time (univariable analysis) in the CLR model was also performed to evaluate whether any risk factors may have been overlooked. All statistical tests were two sided and considered significant at a nominal level of 0.05 (*p* < 0.05). Data were analyzed using IBM SPSS Statistics software (v. 26; IBM Corp., Armonk, NY, USA) and R [28].

### Ethical considerations

The rectal cancer registry in Region Västmanland has ethical committee approval (Uppsala DNR 02-462) and the present study was approved by the ethical committee (Göteborg DNR 2020-04053 and Göteborg DNR 2021-04954).

## Results

Fifty-two patients in the R-LAR and one hundred and four in the O-LAR group were successfully PS matched for age, sex, ASA grade, and tumor level. Only minor differences were found regarding BMI, TNM stage, neoadjuvant chemotherapy, heart disease or diabetes (Table 1). The percentage of patients receiving pre-operative radiation therapy was higher in the O-LAR group (74.0 vs. 46.2%).

**Table 1:** Characteristics of patients who underwent open (O-LAR) and robot-assisted low anterior resection (R-LAR)

Variable	O-LAR	R-LAR
Age, years^a,b^	67 (9.4)	67 (10.9)
Sex (male:female)^a,c^	50:54 (48:52)	25:27 (48:52)
ASA grade^a,b^		
1 or 2	86 (82.7)	45 (86.5)
3	18 (17.3)	7 (13.5)
4	0	0
Tumor distance from anal verge cm^a,b^	9.8 (3.1)	10.2 (2.9)
BMI kg/m^2b^	25.8 (3.5)	26.8 (3.8)
TNM stage^c^		
1 or 2	63 (60.6)	29 (55.8)
3	31 (29.8)	19 (36.5)
4	10 (9.6)	4 (7.7)
Neoadjuvant chemotherapy^b^	19 (18.3)	15 (28.8)
Radiotherapy^b^	77 (74.0)	24 (46.2)
Heart disease^b^	48 (46.2)	22 (42.3)
Diabetes^b^	6 (5.8)	2 (3.8)

^a^Matched variable

^b^Mean (SD)

^c^Number (percentage)

The mean estimated blood loss was 582.7 ml (**± **489.2) after O-LAR and was 86.1 ml (± 67.7) after R-LAR (*p* < 0.001). During the first 24-h post-operative period, 33.7% of the patients in the O-LAR group and 9.6% in the R-LAR group received a blood transfusion (*p* = 0.001). Within the first post-operative 30 days, 32.7% of all patients received a blood transfusion: 43.3% (95% CI 33.6–53.0) in the O-LAR group and 11.5% (95% CI 4.4–23.4) in the R-LAR group (*p* < 0.001).

Within the first 30 days, one patient died in the O-LAR group and none in the R-LAR group. There was no conversion of R-LAR to O-LAR. The rate of surgical site infections postoperatively did not differ significantly between the groups. Anastomotic leakage was found in 9.6% of the patients in the O-LAR group and 17.3% in the R-LAR group (*p* = 0.197). One patient (1.0%) in the O-LAR group and eight (15.4%) in the R-LAR group were re-operated on within 30 days after surgery (*p* = 0.001).

Univariable and multivariable analyses were performed to analyze the risk of transfusion within 30 days (Table 2). Open surgery was significantly associated with the risk of needing a blood transfusion within 30 days after surgery in the univariable analysis [odds ratio (OR) = 6.11, 95% CI 2.35–15.86; *p* < 0.001] and in the adjusted analysis (OR = 4.83, 95% CI 1.74–13.46; *p* = 0.003). Secondary sensitivity analyses of remaining variables showed that lower pre-operative hemoglobin was also significantly associated with higher risk for the need for blood transfusion in the univariable and multivariable analyses.

**Table 2:** Univariable and multivariable logistic regression analyses of the risk of the need for a blood transfusion within 30 days in patients who underwent low anterior resection for rectal cancer

Risk factor	Univariable analysis			Multivariable analysis		
	OR	95% CI	*p* value	OR	95% CI	*p* value
Surgery						
Robotic	1			1		
Open	6.11	2.35–15.86	< 0.001	4.83	1.74–13.46	0.003
BMI	0.96	0.87–1.06	0.403			
Surgery time	0.91	0.71–1.16	0.439			
Neoadjuvant chemotherapy						
No	1					
Yes	1.37	0.56–3.36	0.493			
Neoadjuvant radiation therapy						
No	1			1		
Yes	2.38	1.07–5.31	0.033	1.30	0.50–3.34	0.598
Pre-operative CRP level	1.12	1.03–1.21	0.005			
Iron supplementation						
No	1					
Yes	1.40	0.49–4.02	0.532			
Pre-operative hemoglobin level	0.93	0.90–0.96	< 0.001	0.93	0.90–0.97	< 0.001

## Discussion

Patients who undergo R-LAR have less need for blood transfusion than patients who undergo O-LAR as a result of lower estimated blood loss. A blood transfusion is sometimes necessary, but it should be preceded by a thorough evaluation of the indication, transfusion-related costs, and risk for adverse events such as immunological reactions. In addition, blood transfusion in the peri-operative period may increase infectious and surgical morbidity in the first 30 days after surgery [18, 19, 29, 30] and increases the risk of venous thromboembolism [31]. In this PS-matched cohort of rectal cancer patients who underwent low anterior resection, 43.3% (95% CI 33.6–53.0) of all patients who underwent O-LAR and 11.5% (95% CI 4.4–23.4) who underwent R-LAR received a blood transfusion within 30 days after surgery. Some previous studies have shown higher estimated blood loss and transfusion rates for patients undergoing open colorectal surgery compared with minimally invasive procedures [7, 8, 10] while another study found the blood loss to be comparable between those procedures [9].

In the first 52 consecutive patients who underwent R-LAR, the anastomotic leakage rate was high at 17.3%. This may be explained by the change to a more selective approach of a diverting stoma after the introduction of R-LAR, after which 48% of the patients received a diverting loop ileostomy compared with 85% after O-LAR.

In both the univariable and multivariable analyses, open surgery was significantly associated with the need for blood transfusion within 30 days. Secondary sensitivity analyses showed this to be true for low pre-operative hemoglobin level as well. These variables do not diverge from the experience of clinical praxis and may constitute risk factors.

The mean estimated blood loss for O-LAR (582.7 ml) was higher in the present study than that previously reported [8, 10]. Biffi et al. reported a mean estimated blood loss of 146.4 ml for O-LAR and 83.7 ml for R-LAR [10]. However, the blood transfusion rate from our study is similar as reported in other studies [24, 25, 32–36]. As in other studies, the estimated blood loss in our study was recorded by the anesthesiologist and/or the surgeon in charge, and no precise measurement of blood loss was made. This methodology for measuring blood loss contributed some uncertainty to the results and complicates the comparison of estimated blood loss between studies.

When interpreting our findings, one should keep in mind that all patients were managed in a multidisciplinary setting and all surgeries were performed by highly experienced colorectal surgeons from a single center and should be interpreted cautiously and the robotic surgeries represent the authors’ initial experience. Our study has several limitations worth mentioning. First, this study was an observational study, although most of the data were collected prospectively and a PS matching was performed to reduce confounding bias. Second, the included patients underwent surgery between 2000 and 2020, it is possible that differences between groups would have been smaller with a shorter inclusion period. For instance, there was a difference in the number of patients receiving a diverting loop ileostomy between the groups. As the open surgery during the study period was performed by or under supervision of four colorectal surgeons and robotic surgery by two colorectal surgeons, the risk of differences in blood loss due to different surgical techniques among the surgeons would be minor. However, a change of peri- and post-operative care during the study period is possible and brings uncertainty to our results. Third, the groups were matched on PS strata based on age, sex, ASA grade, and tumor distance from the anal verge, but the groups differed in the use of pre-operative radiotherapy: 74% of patients in the O-LAR group and 46.2% in the R-LAR group reflecting adjustments to guidelines and less pre-operative radiotherapy in rectal cancer. However, this variable was not part of the PS matching and may have affected the result because radiation-induced scarring can lead to greater blood loss during surgery. A previous trial reported that patients who underwent open surgery had significantly greater blood loss if assigned to pre-operative radiotherapy compared with no radiation [37]. In our study, the tumor stage was not part of the matching. In the end, we found no important difference in tumor stage between the two groups. Lastly, the current study was not designed to evaluate the risk factors for the need for blood transfusion outside of O-LAR vs. R-LAR. Therefore, post hoc findings from the secondary analyses should be interpreted with caution, as statistical power may be lacking to detect associations with additional risk factors and there may be potential pitfalls in identifying their origin [38].

In conclusion, the estimated blood loss and need for blood transfusion were lower after robotic low anterior resection for rectal cancer compared with open surgery. Open surgery was shown to be associated with an increased need for peri-operative blood transfusion.

## Data Availability

The data that support the findings of our study are not openly available due to reasons of sensitivity and are available from the corresponding author upon request. Data are located in controlled access data storage at Västmanland Hospital, Västerås.
